# First-time testers in the GetaKit study: conceptualizing new paths to care for gbMSM

**DOI:** 10.1093/heapro/daad029

**Published:** 2023-04-26

**Authors:** Patrick O’Byrne, Lance McCready, Jason Tigert, Alexandra Musten, Lauren Orser

**Affiliations:** School of Nursing, University of Ottawa, Ottawa, ON, Canada; Leadership, Higher & Adult Education, Ontario Institute for Studies in Education, University of Toronto, Toronto, ON, Canada; Leadership, Higher & Adult Education, Ontario Institute for Studies in Education, University of Toronto, Toronto, ON, Canada; Ontario HIV Treatment Network, Ottawa, ON, Canada; School of Nursing, University of Ottawa, Ottawa, ON, Canada

**Keywords:** HIV self-testing, first-time testers, Ontario, Race, youth

## Abstract

When analyzing the data for Ontario, Canada, HIV rates continue to be highest among gay, bisexual and other men who have sex with men (gbMSM). Since HIV diagnosis is a key component of HIV care, self-testing has provided options for allowing this population to access care, resulting in a significant number of first-time testers. Between 1 April 2021 and 31 January 2022, 882 gbMSM participants ordered an HIV self-test through GetaKit. Of these, 270 participants reported that they had never undergone HIV testing previously. Our data showed that first-time testers were generally younger, members of BIPOC (Black, Indigenous and people of color) communities and they reported more invalid test results than those who had tested previously. This suggests that HIV self-testing may be a more successful and appealing component of the HIV prevention armamentarium for this population, but one that is not without its shortcomings as an entry to care.

## INTRODUCTION

In Ontario, HIV transmission is ongoing without much change, with ~14% of persons continuing to be unaware they are HIV-positive as of 2018 (Ontario HIV Epidemiology and Surveillance Initiative [OHESI], 2021a). Additionally, the same groups continue to be affected by HIV. Namely, gay, bisexual and other men who have sex with men (gbMSM) account for over half of new HIV infections per annum in our province; other groups disproportionately affected by HIV include persons who identify as African, Caribbean or Black ethnicities, persons from regions where HIV is endemic, Indigenous persons and persons who use injection drugs (OHESI, 2021b). Not only does this situation persist despite ongoing and innovative testing initiatives, but also it signals that Ontario is below the UNAIDS 95-95-95 targets for 2025 in terms of having 95% of persons in the province diagnosed with HIV and linked to HIV care.

Because research shows that HIV self-testing, compared to clinic and outreach testing, corresponds with more frequent testing, higher rates of first-time testers, elevated positivity rates and better user satisfaction, it has been touted as a strategy to decrease the number of persons with undiagnosed HIV (Flowers et al., 2017; Ontario HIV Treatment Network, 2019; Ortblad and Stekler, 2020). If such findings would be replicated in Ontario, however, was unclear. As such, we launched the GetaKit study (www.GetaKit.ca), which is an online registration, risk assessment and ordering platform for free HIV self-tests in Ontario for persons who qualify based on our risk-screening algorithm. This project used the INSTI® HIV self-test, as this was the only test licensed in Canada during the study period.

While we have published on the implementation, algorithm and pilot uptake of GetaKit (see O’Byrne et al., 2021a,b,c), in this paper we report on the full project dataset regarding first-time testers, focusing on gbMSM, as the group most affected by HIV in Ontario. Our focus here is the comparison of first-time testers and those who reported previous HIV testing, specifically highlighting the similarities and differences in these groups for gbMSM participants. These findings add to the literature about HIV self-testing by showing the characteristics of gbMSM who accessed HIV self-testing through GetaKit—specifically those who did so to undergo a first HIV test—and how such testing can be a tool to increase HIV status awareness and access to care among persons at-risk for HIV.

## METHODS

The GetaKit study was an open cohort prospective observational study in which persons could access our website (www.GetaKit.ca), register using multi-factor authentication, complete an automated HIV risk assessment, and, if eligible based on the risk assessment, order an HIV self-test for either home delivery (via federal mail) or curbside pick-up (at local AIDS service organizations, sexual health clinics, pharmacies and university health promotion offices). GetaKit launched as a pilot study on 20 July 2020, in Ottawa and expanded across Ontario on 1 April 2021. Inclusion and exclusion criteria are listed in Table 1.

**Table 1:** Study inclusion and exclusion criteria

Inclusion criteria	Exclusion criteria
•16 years of age or older •Lives in Ontario •HIV-negative or HIV status unknown	•Bleeding disorder •Taking HIV pre-exposure prophylaxis and doing HIV serology per guidelines •In an HIV vaccine trial •No reported risks for HIV

As the only licensed HIV self-test in Canada, the INSTI® self-test was used. This test is a single-use third-generation antibody qualitative assay that uses 50 mcg of fingerstick blood (BioLytical, 2020). The fingerstick blood is mixed with three solutions (diluent, developer and clarifier) that are separately poured into a flow-through membrane (BioLytical, 2020). Possible results are reactive (positive), non-reactive (negative) and invalid (indeterminate). The INSTI® has a published sensitivity of 99.9% and specificity of 99.5% for HIV detection and a window period of 12 weeks (BioLytical, 2020).

All data from registration and the risk assessment were recorded on the website and could be exported to an MS Excel file for analysis. For this paper, we extracted participant responses to questions about age, employment status, living arrangement, ethnicity, gender, sexual orientation, HIV risk and previous HIV testing (self-testing or serology). We specifically sought to identify if there were significant differences between gbMSM participants who reported versus denied a history of HIV testing. For these analyses, we performed a two-tailed *t*-test for age and chi-square testing for all other variables. A *p*-value of 0.05 was set as significant *a priori*. Because our focus for this paper was to report on the full study dataset, looking at differences in testing practices in gbMSM across Ontario, our analytic period was 1 April 2021 to 31 January 2022.

As part of registration, all participants reviewed and electronically signed a digital consent form. This project was funded by the Ontario HIV Treatment Network (EFP-2020-DC1) and the Research Ethics Board at the University of Ottawa approved this project (H-12-20-6450).

## FINDINGS

During the analytic period (1 April 2021 to 31 January 2022), 882 gbMSM participants ordered an HIV self-test through GetaKit. These participants were on average 33 years old, 92% (*n* = 812/882) identified as cis-male, 5% (48/882) gender non-conforming and 2% (22/882) trans-male. Regarding ethnicity, 44% (389/882) identified as White, 16% (145/882) Black, 14% (127/882) East Asian, 7% (64/882) South Asian, 6% (50/882) Arab, 5% (44/882) Latino and 1% (9/882) Indigenous. Overall, 56% reported being born in Canada and 67% (595/882) reported being employed or retired.

Regarding testing history (see Table 2), 69% (608/882) reported previous testing, 25% (220/882) denied such prior testing and 6% (50/882) were uncertain if they had ever completed HIV testing. Those who denied previous testing were younger than those who reported a history of HIV testing (*p* < 0.001). As well, a larger proportion of White participants reported previous testing, compared to participants who identified as Black, Indigenous or Persons of Color, or those who reported multiple ethnoracial identities (*p* < 0.001). For the purpose of this study, all participants who identified as a non-White ethnoracial identity were categorized as BIPOC. A larger proportion of participants who reported being born outside of Canada were also less likely to have previously been tested, compared to those born in Canada (*p* = 0.01). A smaller number of those who lived alone denied prior testing, compared to those who live with others (*p* = 0.001). Regarding risk practices, a smaller proportion of those who reported engaging in sex work reported no prior HIV testing, compared to those who did not report engaging in sex work (*p* = 0.04) and a smaller proportion of those who reported that their sex partners were HIV-positive and/or used injection drugs and/or were born in HIV-endemic countries had not previously completed HIV testing, compared to those who reported that their partners had none of the aforementioned characteristics (*p* = 0.03). In contrast, there were no differences in the proportion who reported versus did not report prior HIV testing among participants who reported personal injection drug use, who identified as cis- versus trans-gender, who identified as gay or bisexual versus a man who has sex with men or who reported being employed versus unemployed. There were also no differences in the proportion of first-time tests for those who lived in urban versus rural areas.

**Table 2:** Testing history

	First-time testers		Previous testers		Bivariate	
	*N*	%	*N*	%	X^2^	*p*
Testing history						
Previous HIV testing	—	—	608	69%	—	—
No previous HIV testing	220	25%	—	—	—	—
Uncertain	50	6%	—	—	—	—
*Demographic characteristics*						
Sex					0.4501	ns
Cis male	201	91%	564	93%		
Trans	19	9%	44	7%		
Sexual orientation					2.3050	ns
Gay or bisexual	207	94%	552	91%		
Man who has sex with men	13	6%	56	9%		
Ethnicity					16.9300	<0.001
BIPOC	133	64%	273	47%		
White	75	36%	304	53%		
Country of birth					6.4076	0.011363
Canada	111	50%	366	60%		
Abroad	109	50%	241	40%		
Living arrangement					10.2534	<0.001
Lives alone	57	26%	231	38%		
Lives with others	162	74%	376	62%		
Geographic location					0.2292	ns
Urban	174	79%	490	81%		
Rural	46	21%	118	19%		
Employment status					1.9742	ns
Employed	132	82%	435	86%		
Unemployed	29	18%	68	14%		
*Risk practices*						
Sex work					4.0915	0.0431
Engages in sex work	8	4%	46	8%		
Does not engage in sex work	212	96%	562	92%		
Injection drug use					2.5864	ns
Uses injection drugs	12	5%	54	9%		
Does not use injection drugs	208	95%	554	91%		
Risk practices of partners					4.5937	0.03209
Sexual partners at-risk	61	28%	212	35%		
Sexual partners have no risk	159	73%	391	64%		
*Results reporting*						
Self-test result reported					0.0318	ns
Yes	148	67%	403	67%		
No	72	33%	202	33%		

Self-test results were reported by 66% (580/882) of gbMSM participants, with 65% (375/580) of these results being reported as negative, 33% (192/580) as invalid, 2% (11/580) as ‘prefer not to report’ and 0.3% (2/580) as positive. A larger proportion of invalid test results occurred among gbMSM participants who reported being first-time testers (*p* = 0.01).

## DISCUSSION

The results of our study of Getakit users who were first-time testers raise several interesting points for discussion. The fact that of the 882 gbMSM enrolled, nearly all identified as cis-males and more than half BIPOC is worth noting. Also worth noting is the fact that the first-time testers in our study were younger, with a larger proportion being born outside Canada. More first-time testers in our study reported invalid results compared to those who had undergone any previous HIV testing.

Our results lend further credibility to the notion that young gbMSM and individuals belonging to non-White ethnoracial groups encounter a disproportionate number of barriers in accessing traditional routes of HIV testing and care, leading them to explore more unconventional paths of access, which GetaKit may be seen as a part of. Among first-time testers in the GetaKit study, 64% identified as part of the BIPOC community and first-time testers were younger than those who reported a history of HIV testing (*p* < 0.001); our data regarding country of birth and living arrangements is also thought to overlap with these data, as those born outside of Canada have a higher rate of non-testing and younger individuals are more likely to live with others. Within the existing literature, a number of barriers for traditional HIV testing have been identified for these intersecting groups, including a lack of ethno-cultural and age-appropriate care and resources, stigma and discrimination both from peers and clinicians, and limited services being offered in the spaces where they reside (O’Byrne and Watts, 2012; Levy et al., 2014; Silberholz et al., 2017; Mathews et al., 2020). Such barriers have led to an increase in the uptake for HIV self-testing, as shown by Frye in a study of 200-pairs of young Black MSM and transwomen in New York City (2021). Further, this correlates with the findings in a study by Kubicek *et al*., which showed that youth will frequently turn to online services to address their healthcare needs, including access to HIV services, usually due to a mix of convenience, perceived anonymity and lack of access to a trusted clinician (2009; see also Gilbert et al., 2017).

Although these results show promise for conceptualizing self-testing as a panacea for young and BIPOC gbMSM who are reluctant to make use of more traditional, community and hospital-based clinics, such a reading should be met with caution. Yes, self-testing has been positively adopted by gbMSM and has led to an increase in HIV testing overall; however, as we have stated, this is potentially due in large part to the real and perceived shortcomings within the traditional HIV prevention armamentarium. For gbMSM, who exist on the outskirts of Rubin’s charmed circle of sexuality, their ability to interface with the traditional healthcare system is dramatically reduced and they are frequently put into positions where they must find ways to help themselves or risk receiving no help at all (Kinsler et al., 2007; Rubin, 2007; Lindroth, 2021; Kipke et al., 2007). Considering this, the uptake of self-testing can be read as a symptom of a persistent limitation of the current healthcare system, specifically its inability to take into account the sociocultural context of gbMSM who are younger and/or identify as BIPOC.

Given how our own results fit into, and bolster, the existent literature on young gbMSM and BIPOC individuals’ difficulty accessing traditional avenues for HIV testing, alternate strategies, including more robust professional learning for clinicians on anti-bias and creating culturally safe services and more concerted efforts to link these populations with non-stigmatizing in-person care, are needed. The high rate of invalid tests reported among first-time testers (*p* = 0.01) further highlights this need, indicating that a significant portion of the individuals who made use of GetaKit did not in fact discover their HIV status due to a range of possible reasons, including test malfunction or not doing the test correctly. This is especially concerning given trends identified within the existent literature, noting that many gbMSM who have never undergone HIV testing in the past or who have experienced invalid test results, assume they are HIV-negative, even if they frequently take part in behaviors that are considered a high-risk for HIV transmission. For example, a study by Alexovitz *et al.* of 2275 Black, Hispanic and White gbMSM found that of the 471 individuals who had never undergone an HIV test, 57% reported having regular condom-less sex with casual partners and yet self-perceived as having no possibility of being HIV-infected and reported their status as HIV-negative to potential partners (Alexovitz et al., 2017; see also Choi et al., 2018). While this is not particularly surprising given what we know about how HIV and chronic illness diagnoses forces an ontological shift concerning one’s self-perception, self-worth and the ways in which they interact with society, it highlights the need for these individuals to receive additional, in-person supports and education (Dowshen et al., 2009; Wolitski et al., 2009; Gupta and Sudhesh, 2019). In relation to our own data and the large number of invalid tests reported, this means that these respondents may be putting others at heightened risk for HIV transmission. Further, due to the nature of self-testing, in that it is done alone, within a private setting, and without the presence of a clinician, GetaKit cannot ensure that users are receiving information about treatment options, counseling, PEP and PrEP that can best address their sexual health needs.

Though there are areas of concern regarding the high number and composition of the first-time tester sample, our data also demonstrate GetaKit’s potential. As noted in the literature, young gbMSM (under 30 years old) are an incredibly at-risk group, making up a significant portion of all new HIV cases while also experiencing very low attrition rates regarding regular in-person testing and linkage to care (Lessard et al., 2017). Given this, GetaKit, via its online medium, may be seen to increase access to care for this group. Relatedly, our data also suggest that GetaKit may be a viable option, at least in principle, for reaching new Canadians, with 50% of respondents being born abroad. Considering the high number of barriers to access healthcare for new immigrant populations, and the large number of people whom GetaKit was made available to despite any dedicated outreach, self-testing may be particularly effective in addressing the needs of this underserviced group if a more coordinated effort is made. Finally, the data suggest that GetaKit may be especially useful in overcoming one of the greatest systematic barriers to HIV testing: geography. Although most results were reported from urban areas, it is noteworthy that there was no measurable difference between first-time testers and previous testers in rural areas; this may be taken to suggest that GetaKit was viewed as an easily accessible, and thus readily used, method for HIV testing within more traditionally inaccessible rural communities. Contrary to this, we also recognize that these data may be interpreted to indicate that GetaKit was not in fact reaching those most at-risk in rural communities—individuals who have never been tested—and that more research is needed into this phenomenon.

## LIMITATIONS

The data presented here must be analyzed with certain limitations in mind. First, these results are based on self-reporting, with about 38% of the results not having been reported. Second, the data were collected during the COVID-19 pandemic which may have prompted some individuals who were first-time testers to use GetaKit not because they favored the online medium, but because access to more traditional testing routes was severely limited during this period. Relatedly, while we asserted that the online medium was appealing to younger and racialized populations based on the existing literature and that this was part of their reason for accessing GetaKit, this is purely speculative in nature, with further research being needed in this area. Finally, although it has been previously noted that there was no concerted effort to reach new Canadian populations, there were efforts made to target Black populations through partnerships with organizations that specifically service this group. There is room for GetaKit to partner with healthcare organizations and programs serving other BIPOC groups disproportionately impacted by HIV, specifically Indigenous populations. The need for collaboration with Indigenous organizations may also explain why gbMSM who identify as Indigenous are underrepresented in our sample, despite being a population that is disproportionally impacted by HIV in Canada (Ontario HIV Treatment Network, 2019b). While our work has shown that GetaKit and self-testing has amazing potential within the Canadian context, it continues to need further refinement before it can be hailed as an unequivocable success.

## CONCLUSIONS

In this paper, we have highlighted the demographic characteristics of first-time testers who took part in the first 10 months of the Ontario-wide GetaKit study. Our findings illustrated that first-time testers were, on average, younger than repeat testers, more likely to be members of a racial/ethnic minority population, and that they reported invalid test results more frequently than repeat testers. While it is beyond the scope of this study to conclusively answer why these groups would choose self-testing over more traditional, in-clinic, methods of HIV testing, it nevertheless provides a response to our initial query regarding whether GetaKit would be taken up by gbMSM as a tool to increase their HIV status awareness and access care, in Ontario, amidst the COVID-19 pandemic, in ways that mirrored previous studies. In this regard, our study would evidence that gbMSM in Ontario, specifically those who are members of traditionally underserviced communities, do view self-testing as a means to glean their HIV status and interact with the HIV care continuum.

While these finding give credence to the fact that self-testing can be an important part of the HIV care continuum for young gbMSM, racialized communities and new Canadians, this paper has only begun to scratch the surface in terms of coming to an understanding as to why and how these communities conceptualize and interact with self-testing as part of the HIV prevention armamentarium. Although this study demonstrates the ability of self-testing to reach individuals who may have otherwise gone untested, an action that should be seen as positive for both the individual and society, it does not tell us why these same individuals were not being tested in the first place, or why they came to see self-testing as a viable option. To investigate this, it is recommended that further qualitative research be done, in order to gain a better understanding, through personal narratives, of the decision-making/thought processes of first-time testers. Further, while it is widely accepted that the first link within the HIV continuum of care is testing, ensuring that individuals are given access to information about treatment options, counseling, PEP and PrEP, the fact that so many first-time testers reported inconclusive results essentially renders their interactions with the continuum of care as moot; for all intents and purposes the individual remains untested and has acquired no more knowledge about their own HIV status or how to access other sexual healthcare options. To better understand and address this phenomenon, it is recommended that further research be done into why such a high number of first-time testers have invalid test results, specifically research that can lead to practical changes regarding how the INSTI® HIV self-test is packaged and distributed for use, as well as to further theorize on how self-testing fits into a pathway of care whose concept far pre-dates its use. Although self-testing is an amazing tool with great potential for reaching traditionally underserviced communities, it is imperative that the ways in which people interact with it, whether as a first-time testers or repeat user, and their motivations for doing so, receive further investigation.
